# Investigation of Stress Distribution and Fatigue Performance in Restored Teeth Using Different Thicknesses of Adhesive Materials and Different Restorative Materials: 3D Finite Element Analysis (FEM)

**DOI:** 10.3390/ma18163888

**Published:** 2025-08-20

**Authors:** Reza Mohammadi, Sinem Alkurt Kaplan, Abdulkadir Harmankaya, Hakan Yasin Gönder

**Affiliations:** 1 Faculty of Dentistry, Necmettin Erbakan University, Konya 42090, Turkey; 2 Department of Oral and Maxillofacial Diseases, Faculty of Medicine, University of Helsinki, 00014 Helsinki, Finland; 3 Department of Restorative, Faculty of Dentistry, Necmettin Erbakan University, Konya 42090, Turkey; sinem.alkurt@gmail.com (S.A.K.); abdulkadirharmankaya@gmail.com (A.H.); hygonder@erbakan.edu.tr (H.Y.G.)

**Keywords:** adhesion, restorative materials, stress distribution, fracture lifetime, restoration

## Abstract

**Background:** This study aimed to compare the stress distribution and fracture resistance of dental tissues and restorative materials with varying adhesive layer thicknesses and different restorative materials. **Methods:** A caries-free mandibular first molar (tooth #36) was scanned using CBCT. The scanned files were processed in Mimics 12 software for segmentation of enamel, dentin, and pulp tissues and then exported to STP format. Cavity preparations (DO, MO, MOD, and O) were designed in SolidWorks 2023. Bulk-fill composite, conventional composite, and hybrid composite were used for restorations with adhesive layers of 10, 15, and 20 μm thick. Stress distribution and fracture resistance were analyzed using 3D finite element analysis. **Results:** The highest stress values in enamel, dentin, and adhesive material were observed in models restored with bulk-fill composite, while the highest stress values within the restoration were found in models restored with hybrid composite. As the adhesive layer thickness decreased, stress accumulation within the restorative material increased. Enamel fractures occurred first in models with bulk-fill composite. Among restorative materials, fractures initiated first in models restored with hybrid composite, while the latest fracture onset was observed in models with bulk-fill composite. **Conclusions:** Restorative materials with low Young’s modulus cause excessive stress accumulation in enamel and dentin, leading to early fracture of these tissues. In contrast, materials with a high Young’s modulus transfer more stress to the restoration, causing premature fracture of the restorative material.

## 1. Background

Dental caries remains one of the most common and preventable oral diseases, with individuals being susceptible to its development throughout their lifetime. If left untreated, caries can lead to toothache or eventual tooth loss [1,2]. One of the most critical properties of restorative materials, which are widely used and continuously developed in dentistry, is their resistance to masticatory forces. The physical, chemical, and mechanical characteristics of widely used restorative materials play a crucial role in determining how stress is distributed within dental tissues and directly impact the overall longevity of restorations. Minimal stress accumulation within both the tooth structure and restorative material is considered an ideal outcome. However, achieving low stress accumulation in both dental tissues and restorative materials simultaneously is often challenging [3,4].

Dental caries can be classified based on the location and extent of the lesions resulting from demineralization of the enamel and dentin tissues. Among the various classification systems, the most widely used and historically influential is the system developed by G.V. Black. This system categorizes carious lesions according to their anatomical location on the tooth and has served as a foundational reference in clinical dentistry for decades. In this classification, lesions located in the pits and fissures of the occlusal surface are designated as Class I, while those on the proximal surfaces (mesial or distal) of posterior teeth are categorized as Class II. When a carious lesion progresses and leads to significant loss of tooth structure, restorative intervention becomes essential. In contemporary restorative dentistry, particularly in posterior teeth, resin-based composite materials have become the preferred choice due to their favorable biomechanical and aesthetic properties. These composites offer a tooth-colored appearance, micromechanical bonding to tooth structures, and the absence of mercury, making them a widely accepted alternative to dental amalgam. The selection of an appropriate restorative material is guided by both the clinical characteristics of the lesion and the patient’s aesthetic expectations [5].

Bulk-fill composite resins, owing to their advanced physical properties such as aesthetic integration, mechanical strength, and wear resistance, are among the most commonly preferred polymer-based restorative materials for the direct restoration of lost tooth structure in posterior regions. When used in conjunction with modern adhesive systems, these materials are considered suitable restorative options that can successfully mimic the characteristics of natural dental tissues [6]. The clinical success of resin composite restorations is influenced by the properties of the material used and the associated application technologies. The mechanical strength, wear resistance, and aesthetic properties of composite resins are significantly influenced by their composition, particularly the type and size of filler particles. The ability to replicate natural tooth color is essential for aesthetic success and patient satisfaction. Moreover, the choice of adhesive system plays a critical role in determining the longevity and retention of the restoration. Handling properties such as flowability, viscosity, and ease of manipulation are key for the accurate placement and adaptation of the material. Lastly, the polymerization mechanism affects cure depth, degree of conversion, and bond strength to dental tissues [7]. Hybrid composite materials are multifunctional materials used in dentistry to provide multiple functional advantages, such as mechanical strength and/or conductivity, while also meeting aesthetic requirements. These materials are particularly ideal for dental restorations as they mimic the natural properties of teeth, offering both aesthetic appeal and functional durability. In dentistry, especially for posterior restorations, such hybrid materials offer durable and aesthetically pleasing results, enabling long-lasting restorations that meet both functional and cosmetic demands [8].

The clinical longevity of adhesive dental restorations is largely dependent on the durability of the bond between the restoration and the tooth. Polymerization shrinkage of dental composites generates contraction stress on the adhesive interface once the resin composite is light-cured. This can cause the bond to detach from the tooth substrate, especially under high-C-factor conditions. Additionally, factors such as the moist oral environment, thermal fluctuations, acid exposure, biofilm accumulation, and chewing forces continuously challenge the restoration-tooth bond during functional use, potentially compromising its durability [9]. Recent advances in restorative dentistry have largely focused on perfecting the bonding process and adhesive interfaces [10]. Modern adhesives have been successful in reducing the steps necessary to achieve a strong adhesive bond. However, despite these advancements, the inability of adhesive agents to polymerize in the presence of oxygen remains an issue. This limitation can result in the formation of an oxygen-inhibited layer on the surface, which can reach up to 40 μm in thickness [11]. Consequently, when excessive air thinning is applied, the adhesive layer may become thin enough to prevent full polymerization. Magne (2006) recommended the application of a thick adhesive layer, ranging from 60 to 300 microns, to ensure adequate polymerization depth and protect the hybrid layer after the unpolymerized surface layer is removed [12]. The adhesive interface is crucial in stress transmission between the restoration and the tooth, and its thickness affects both the mechanical performance and the durability of the bond. Clinically, thin adhesive layers are preferred to reduce polymerization shrinkage and interfacial stress, and studies using scanning electron microscopy (SEM) have reported typical adhesive film thicknesses ranging between 10 and 20 µm. For example, Kharouf et al. [13] observed adhesive layers with mean values between 10 ± 4 µm and 17 ± 9 µm among several universal adhesives, aligning with manufacturer expectations and clinical protocols. Similarly, Ausiello et al. [14] incorporated 10 and 30 µm thick adhesive layers in their FEA study to simulate bonded restorations, highlighting the relevance of such values in modeling realistic conditions.

Various stress distribution analysis methods are employed in dentistry, with the most widely used being the Finite Element Stress Analysis (FEA) method. This technique was developed by Hrennikoff and Courant to analyze stress distribution in complex models [15]. Originally developed for engineering applications, this analysis method has been increasingly utilized in dentistry. Determining the stress magnitudes and distribution patterns in living tissues under functional loads is a complex, time-consuming, and costly process. Consequently, finite element analysis (FEA) has emerged as a standard computational technique in contemporary dental research for assessing stress patterns in biomimetic models of dental structures [16]. The finite element analysis (FEA) method has provided researchers with the opportunity to conduct non-invasive analyses in a computerized environment, without the need for living tissues [17]. In addition to its many advantages, this analysis method has certain limitations, including the challenges of transferring living tissues into a computerized environment using software, the potential for human error, the requirement for experienced engineers, and the need for extended computation times [18].

The aim of this study is to evaluate and compare the stress distribution patterns in dental tissues and restorative materials when restored with different materials, along with the corresponding fracture resistance. In this study, a single molar tooth model was used to ensure standardization and improve the comparability of results. Individual anatomical differences and varying tooth morphologies can introduce unnecessary variability in stress distribution outcomes; therefore, a controlled modeling approach was preferred. Similar modeling strategies have been widely applied in finite element studies, as single-tooth models allow for more focused and reliable evaluation of material properties and adhesive factors without the additional complexity of multi-tooth simulations.

To date, many studies have investigated the biomechanical behavior of restored teeth using various restorative materials and cavity designs. However, most of these studies have focused either only on material properties or cavity configuration, without evaluating the combined effect of adhesive layer thickness. The novelty of this study lies in its comprehensive approach, simultaneously analyzing the influence of both adhesive thickness and restorative material stiffness on stress distribution and fatigue performance in Class I and Class II cavities through 3D finite element analysis. By incorporating these two clinically relevant variables together, our study provides a more realistic simulation of clinical conditions and offers practical guidance for optimizing adhesive protocols and material selection in restorative dentistry. The null hypothesis is that variations in the mechanical properties of restorative materials lead to differences in stress accumulation within both the tooth structure and the restoration, which in turn influence the overall fracture life of the system.

## 2. Methods

A sound mandibular first molar (tooth #36), free of caries and cavitations, structural defects, and loss was imaged using cone-beam computed tomography (CBCT). This study was unanimously approved by the Ethics Committee of Necmettin Erbakan University, Faculty of Dentistry, with the application ID: 23544. The scanning was performed within a cylindrical field of view measuring 40 mm in both diameter and height, centered at the X-ray source’s rotational axis. The imaging parameters included a current of 5 mA (milliampere, the electric current value) and a peak tube voltage of 90 kVp (peak voltage, the highest voltage of the X-ray tube). The acquisition was conducted with a 160 µm voxel size and an exposure duration of 17.5 s.

The acquired DICOM images were imported into Mimics software (version 12.00, Leuven, Belgium) for the segmentation process. Within the software, enamel, dentin, and pulp tissues were individually segmented and exported into STL files. The STL files were then imported into Geomagic Design X for surface optimization and saved as STP files. The optimized 3D tooth model was later positioned within a simulated structure representing cortical and cancellous bone using Solidworks 2023 software program (Solidworks Corp., Waltham, MA, USA). A 0.2 mm thick periodontal ligament was designed around the tooth in the final models.

**Figure 1:**
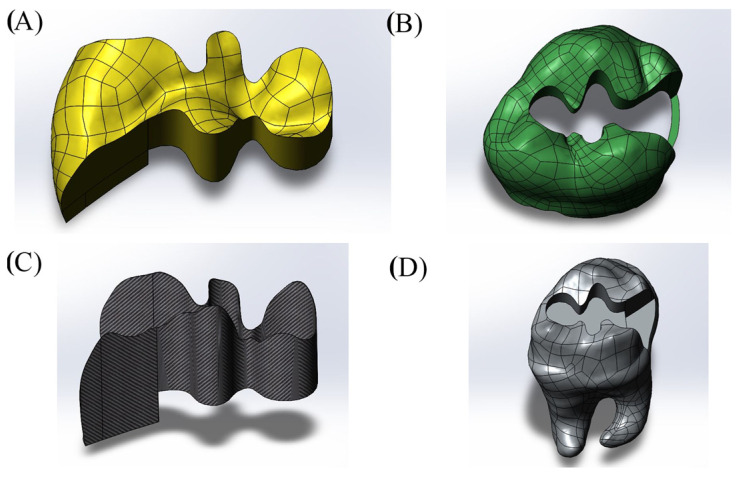
Model with Class II disto-occlusal (DO) cavity design. (**A**) Restoration, (**B**) enamel, (**C**) adhesive material, (**D**) dentin.

**Figure 2:**
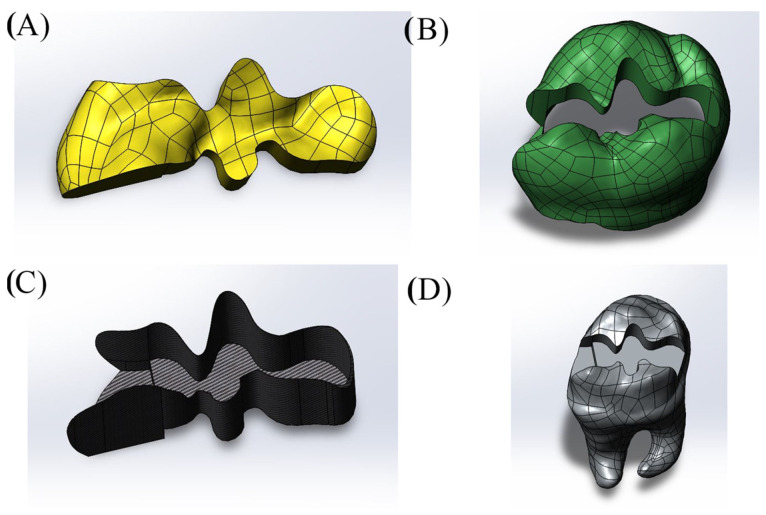
Model with Class II mesio-occlusal (MO) cavity design. (**A**) Restoration, (**B**) enamel, (**C**) adhesive material, (**D**) dentin.

**Figure 3:**
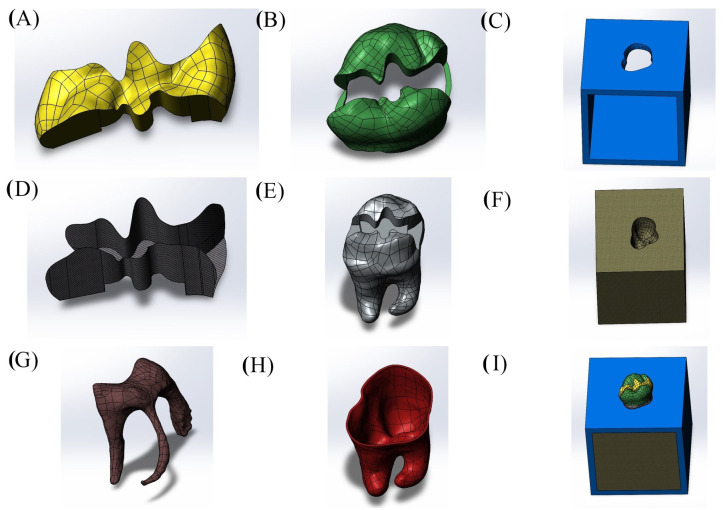
Class II model with mesio-occluso-distal (MOD) cavity design. (**A**) Restoration, (**B**) enamel, (**C**) cortical bone, (**D**) adhesive material, (**E**) dentin, (**F**) spongiose bone, (**G**) pulp, (**H**) periodontal ligament, (**I**) completed whole model.

**Figure 4:**
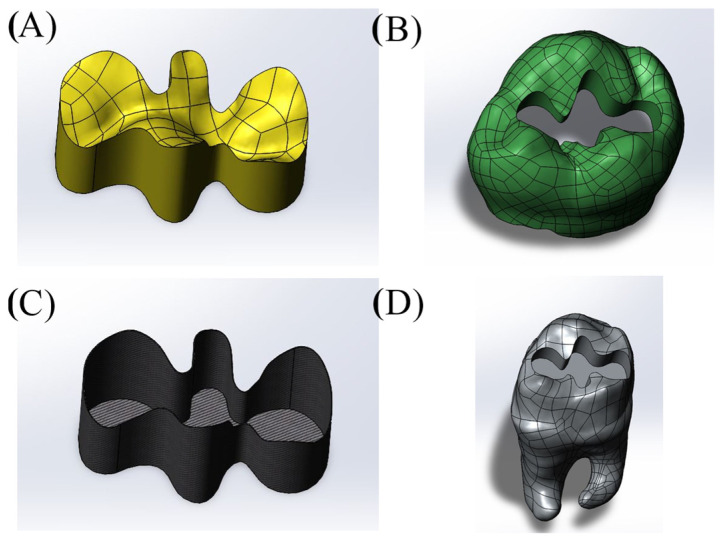
Model with Class I occlusal (O) cavity design. (**A**) Restoration, (**B**) enamel, (**C**) adhesive material, (**D**) dentin.

Cavity designs were created on the final tooth model based on Class II mesio-occlusal (MO), disto-occlusal (DO), mesio-occluso-distal (MOD) and Class I occlusal (O) preparation standards. Additionally, adhesive material layers with thicknesses of 10, 15 and 20 µm were applied to these models; these thicknesses were selected based on previous studies, as these values reflect clinically relevant and commonly observed ranges as presented in Figure 1, Figure 2, Figure 3 and Figure 4 [13,14].

The restorative materials selected and evaluated in this study were bulk-fill composite, conventional composite and hybrid composite resin. These final designs were then exported in STL format to be utilized in finite element analysis (FEA). To ensure accurate simulation within the digital environment, it was essential to define the mechanical properties of both dental tissues and restorative materials; these values are provided in Table 1.

**Figure 5:**
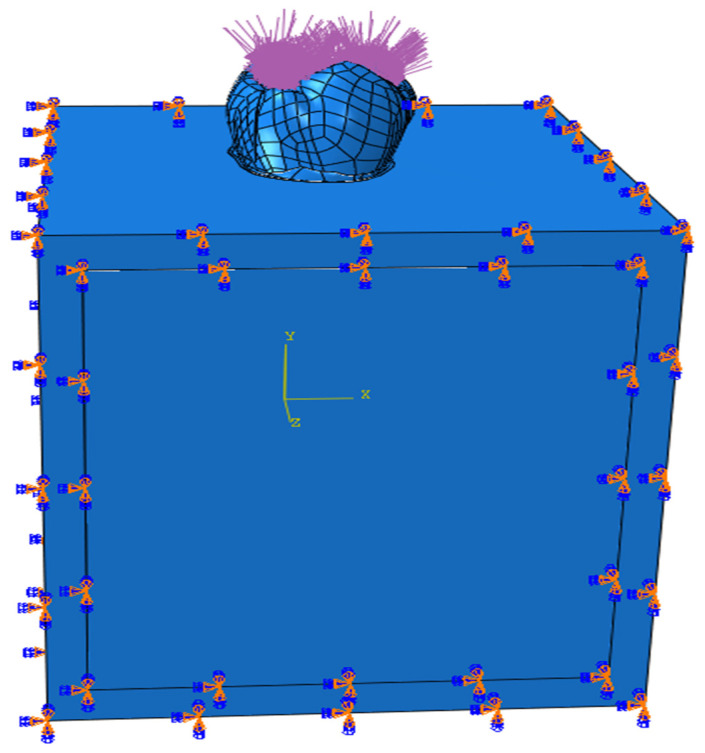
Loads acting on the tooth surface and boundary conditions.

Finite element analysis was performed using Abaqus software (2020, Dassault Systèmes Simulia Corp., Johnston, RI, USA). In the simulation, each component was assigned specific material characteristics. The jawbone was constrained in all directions with respect to motion and rotation. A vertical occlusal force of 600 Newtons (N) was applied to the model throughout the simulation to replicate physiological masticatory loading conditions. Load distribution and boundary condition settings are displayed in Figure 5. The resulting stress distribution was recorded as Von Mises stress values.

The magnitude of force applied to the tooth model was based on average fatigue thresholds reported in prior in vitro studies involving restored molars. To evaluate the fatigue performance of the restorative materials as well as the enamel and dentin, the stress values obtained during loading were compared against a stress–life (S–N) curve based on maximum principal stress. This curve describes the relationship between stress amplitude (*σ*_0_) and the number of cycles to failure (N).

Fatigue parameters for the restorative materials were derived from three-point bending tests, consistent with methods used in previous research. The fatigue response of each material was then estimated using the non-linear Basquin equation, expressed as “$\sigma_{a}=A{\left(N\right)}^{B}$” [27].

The A and B coefficients used in the Basquin equation for the restorative materials are listed in Table 2. The Wöhler (S–N) curves for enamel and dentin were derived from previously published data and were plotted under fully reversed loading conditions (*σ_m_* = 0). All other mean stresses (*σ_m_* ≠ 0) are mathematically represented by the equation: “$\sigma_{a}=(\sigma_{f}-\sigma_{m}){\left(2N\right)}^{b}$” [14,28].

## 3. Results

### 3.1. Results on Stress Distribution

#### 3.1.1. Results of the Model with Class II Disto-Occlusal Cavity

In restorations of Class II disto-occlusal cavities using bulk-fill composite resin, enamel and dentin showed the highest stress levels when the adhesive layer was 15 µm thick, whereas the lowest stress concentrations were observed with a 10 µm thick adhesive layer. Conversely, for both the restoration and adhesive materials, the greatest stress value was noted in the model with a 10 µm thick adhesive layer, while the lowest stress was found in the model with a 20 µm adhesive thickness.

When conventional and hybrid composite resins were applied to similar cavity designs, the enamel and dentin again showed peak stress values in models with a 15 µm thick adhesive layer, and minimum stress in those with a 10 µm adhesive thickness. For the restorative and adhesive parts, the highest stress appeared in the 10 µm thick adhesive material, while the lowest stress values were noted in models with 15 µm adhesive thickness for the restoration and 20 µm for the adhesive material.

**Figure 6:**
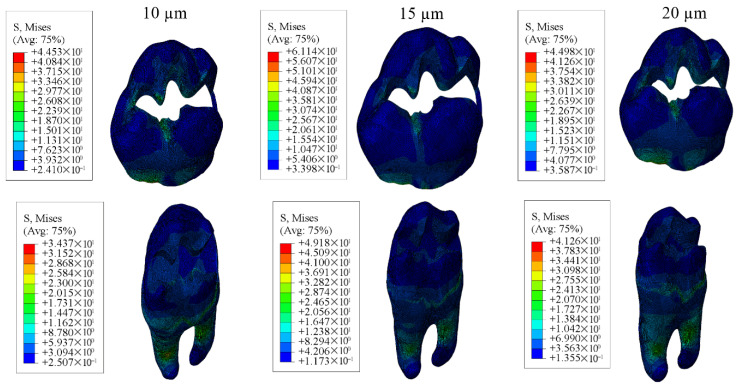
Stress distribution regions in enamel and dentin tissues of the models in which bulk-fill composite resin was used.

**Figure 7:**
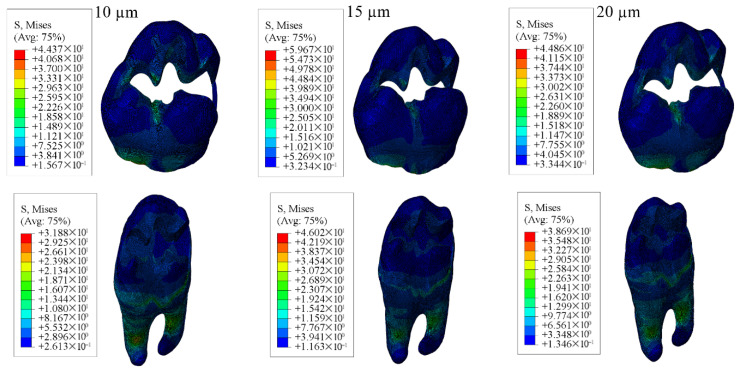
Stress distribution regions in enamel and dentin tissues of the models using conventional composite resin.

**Figure 8:**
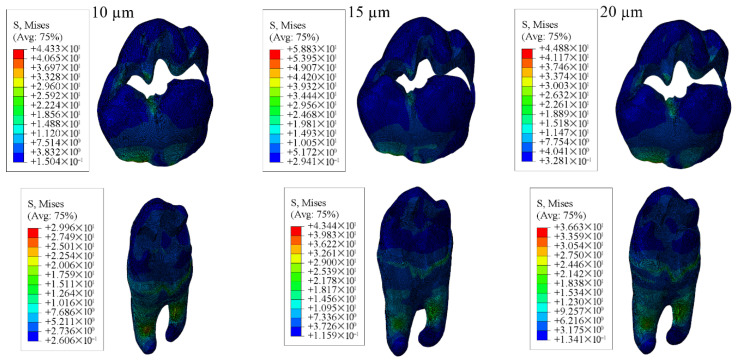
Stress distribution regions in enamel and dentin tissues of models using hybrid composite resin.

**Figure 9:**
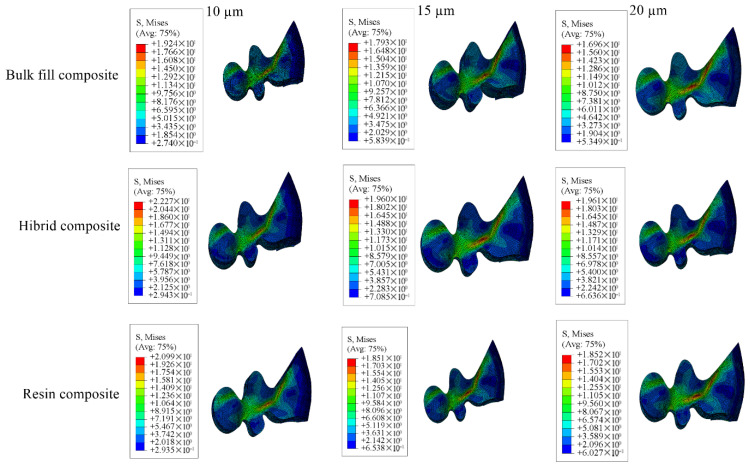
Stress distribution regions in restorative materials of a model with a Class II disto-occlusal cavity.

**Figure 10:**
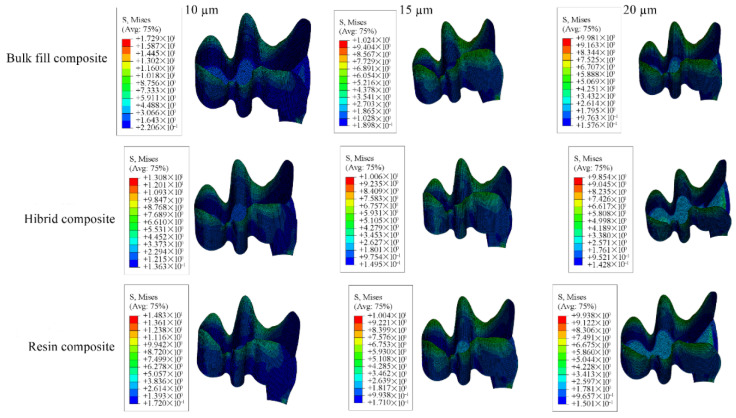
Stress distribution regions in adhesive materials of a model with a Class II disto-occlusal cavity.

Detailed stress magnitudes are listed in Table 3, and stress distribution zones are illustrated in Figure 6, Figure 7, Figure 8, Figure 9 and Figure 10.

Among all configurations, the use of bulk-fill composite resin resulted in the highest stress values in the enamel, dentin, and adhesive layers. In contrast, the greatest stress concentration in the restorative material itself was observed in the models with hybrid composite resin. Furthermore, a reduction in adhesive layer thickness was associated with an increase in stress accumulation within the adhesive material.

#### 3.1.2. Results of the Model with Class II Mesio-Occlusal Cavity

When Class II mesio-occlusal cavities were restored using bulk-fill, conventional, and hybrid composite resin materials, the highest stress values in the enamel were observed in models with 10 µm adhesive layer, while the lowest values occurred in models with a 15 µm adhesive thickness. In the dentin, the highest stress values were observed in models with a 20 µm adhesive layer, whereas the lowest were found with 15 µm thickness.

**Figure 11:**
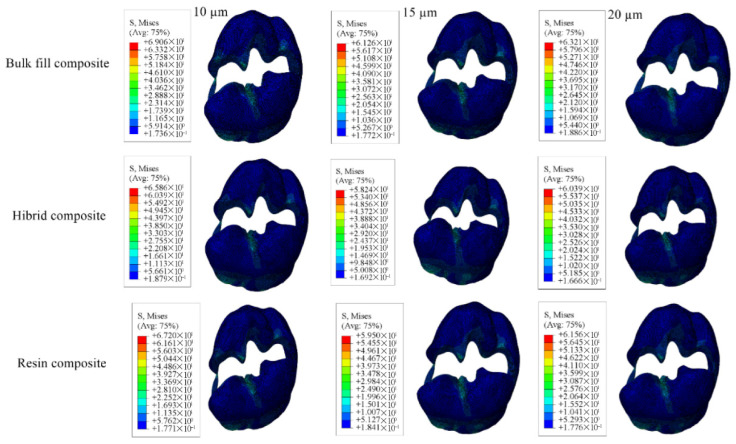
Stress distribution regions in enamel tissues of a model with a Class II mesio-occlusal cavity.

**Figure 12:**
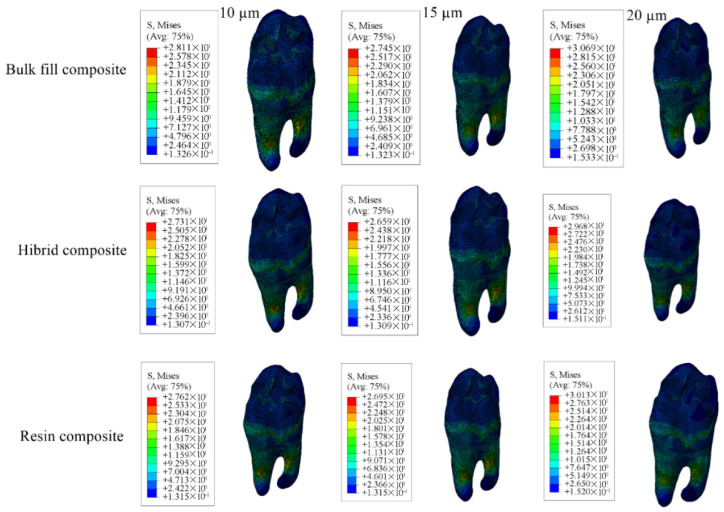
Stress distribution regions in dentin tissues of a model with a Class II mesio-occlusal cavity.

**Figure 13:**
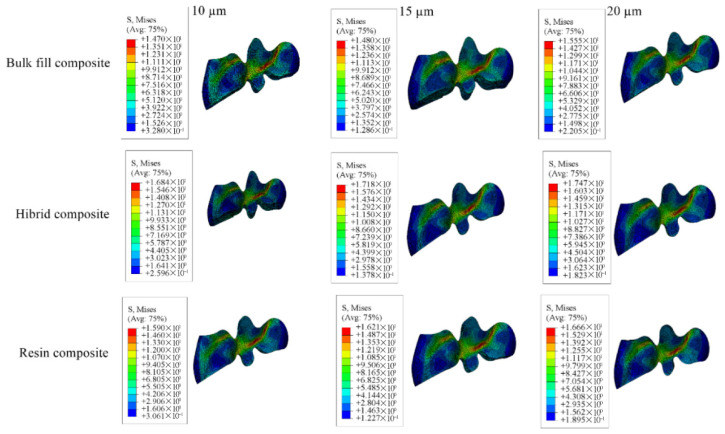
Stress distribution regions in restorative materials of a model with a Class II mesio-occlusal cavity.

**Figure 14:**
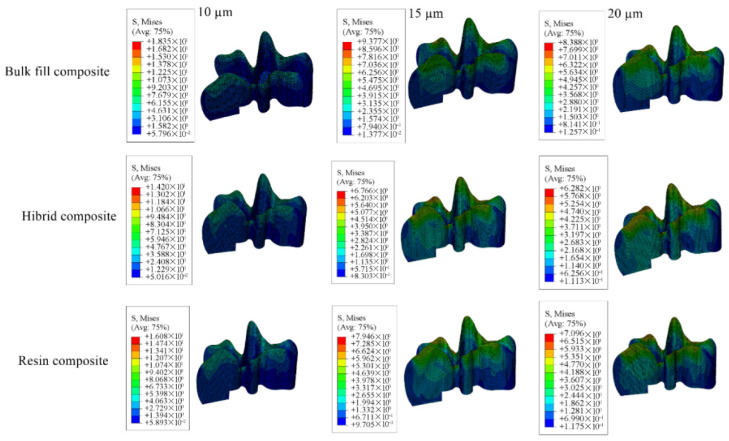
Stress distribution regions in adhesive materials of a model with a Class II mesio-occlusal cavity.

Concerning the restorative material, stress peaked in models with a 20 µm adhesive layer and was lowest in those with 10 µm. For the adhesive material itself, the highest stress was observed in models with 10 µm thickness and the lowest with 20 µm thickness. The corresponding stress values are presented in Table 4, and the stress distribution patterns are shown in Figure 11, Figure 12, Figure 13 and Figure 14.

When each adhesive layer thickness was analyzed separately, models with bulk-fill composite resin showed the highest stress values in enamel, dentin, and adhesive material. In contrast, the highest stress within the restorative material was noted in models restored with hybrid composite resin. Moreover, thinner adhesive layers were associated with increased stress accumulation within the adhesive.

#### 3.1.3. Results of the Model with Class II Mesio-Occluso-Distal (MOD) Cavity

When Class II mesio-occluso-distal cavities were restored using bulk-fill, conventional, and hybrid composite resin materials the highest stress in enamel was observed in models with a 10 µm thick adhesive layer, while the lowest stress occurred in models with a 20 µm thick adhesive layer. In dentin, stress distribution was highest in models with 20 µm adhesive thickness and lowest in those with 15 µm thickness.

**Figure 15:**
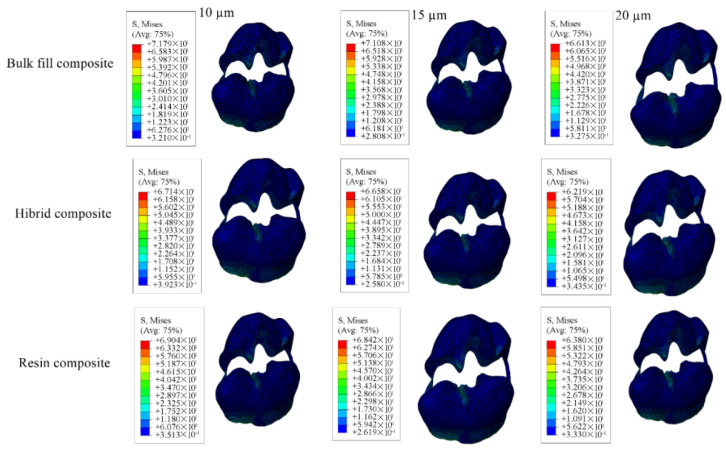
Stress distribution regions in enamel tissues of a model with a Class II mesio-occluso-distal (MOD) cavity.

**Figure 16:**
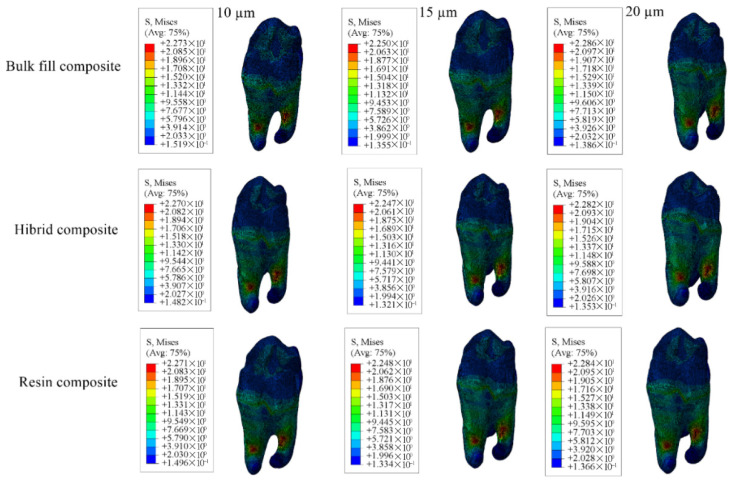
Stress distribution regions in dentin tissues of a model with a Class II mesio-occluso-distal (MOD) cavity.

**Figure 17:**
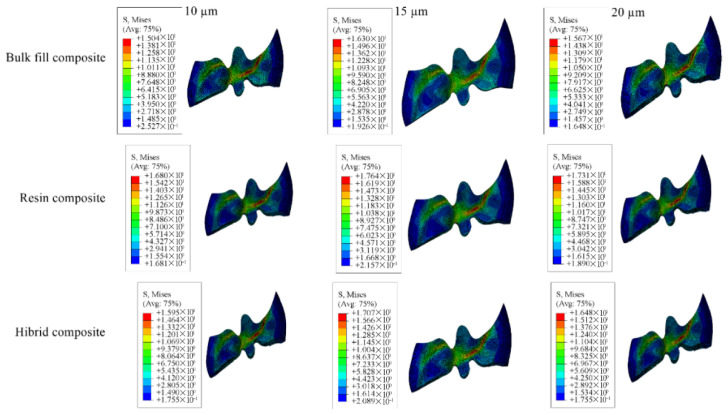
Stress distribution regions in restorative materials of a Class II mesio-occluso-distal (MOD) cavity model.

**Figure 18:**
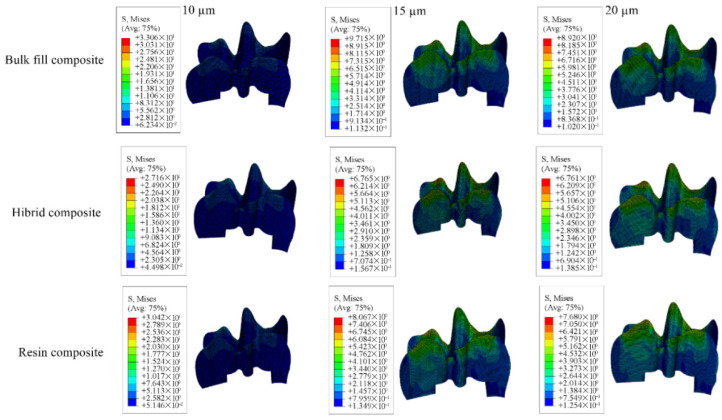
Stress distribution regions in adhesive materials of a model with a Class II mesio-occluso-distal (MOD) cavity.

For the restorative material, the greatest stress was found in models using 15 µm adhesive layer, whereas the lowest was noted in those using 10 µm. Stress within the adhesive material itself was highest in the models with 10 µm adhesive thickness and the lowest in the 20 µm. Numerical results are shown in Table 5, and the stress distribution pattens are shown in Figure 15, Figure 16, Figure 17 and Figure 18.

When each adhesive thickness group was examined individually, models with bulk-fill composite resin showed the highest stress levels in enamel, dentin, and adhesive layers. In contrast, the hybrid composite resin group showed the highest stress within the restorative material. Furthermore, as the thickness of the adhesive material decreased, the stress values accumulated in the material increased.

#### 3.1.4. Results for the Model with Class I Occlusal Cavity

When Class I occlusal cavities were restored using bulk-fill, conventional, and hybrid composite resins, the highest enamel stress was observed in models with a 15 µm thick adhesive layer, while the lowest was observed in those with a 20 µm thick layer. In dentin, the maximum stress was noted in models with 20 µm thick adhesive layer and the minimum in models with a 15 µm thick adhesive layer.

For restorations with bulk-fill composite resin, the highest stress in restorative material occurred in models with a 10 µm thick adhesive thickness and the lowest in models with 15 µm thickness.

**Figure 19:**
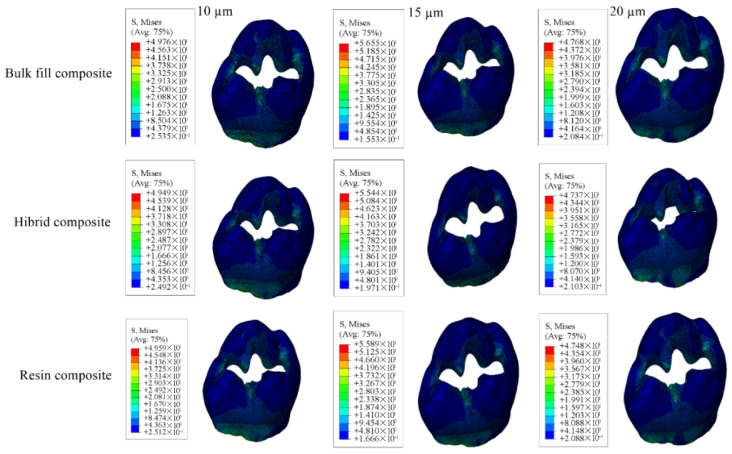
Stress distribution regions in enamel tissues of a model with a Class I occlusal cavity.

**Figure 20:**
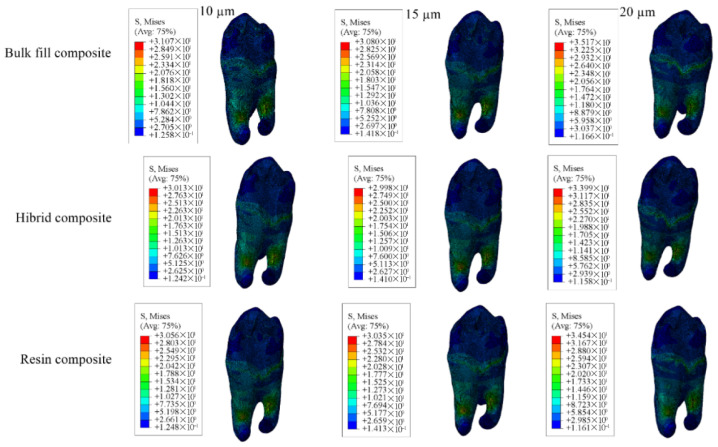
Stress distribution regions in dentin tissues of a model with a Class I occlusal cavity.

**Figure 21:**
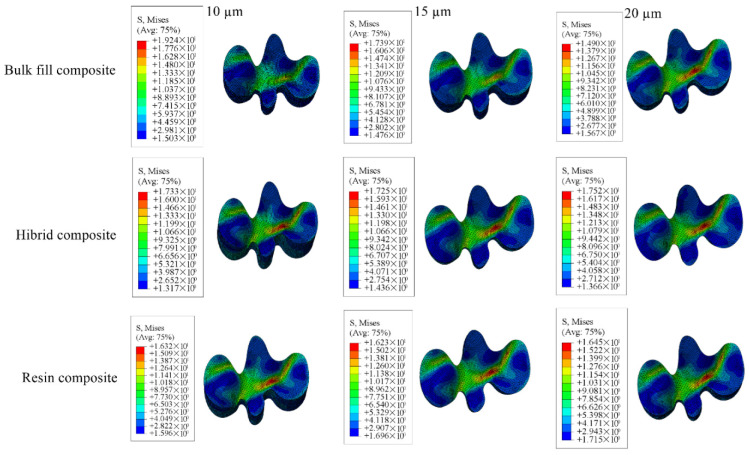
Stress distribution regions in restorative materials of a model with a Class I occlusal cavity.

**Figure 22:**
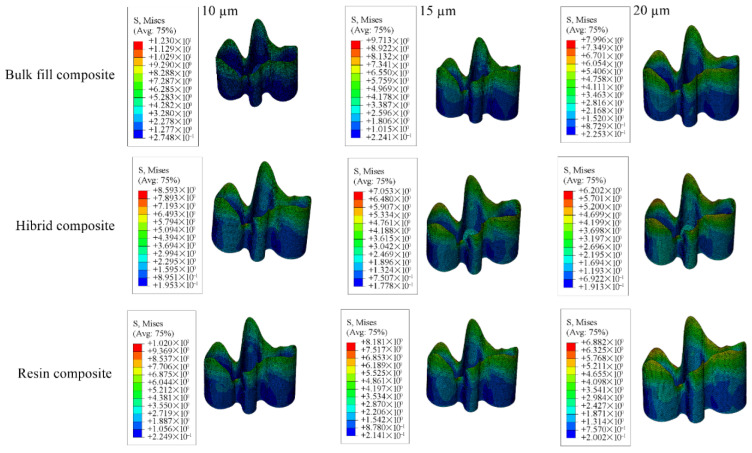
Stress distribution regions in adhesive materials of the model with Class I occlusal cavity.

The adhesive material itself showed the greatest stress accumulation in models with 10 µm adhesive thickness and the least in those with 20 µm adhesive thickness. Numerical values are listed in Table 6, and corresponding stress areas are shown in Figure 19, Figure 20, Figure 21 and Figure 22.

In models with 10 µm and 15 µm adhesive thicknesses, the highest stress values across all tissues and materials were recorded in those restored with bulk-fill composite resin. In models with 20 µm thick adhesive layer, bulk-fill composite resin still produced the highest stress in enamel, dentin, and adhesive, while hybrid composite resin showed the highest stress within the restorative material. Overall, thinner adhesive layers were associated with increased stress in adhesive material.

### 3.2. Results of Fracture Lifespan

#### 3.2.1. Results of the Model with Class II Disto-Occlusal Cavity

When Class II disto-occlusal cavities were restored using bulk-fill, conventional, and hybrid composite resins, and each group was evaluated separately, the highest number of cycles until enamel fracture was recorded in the model with 10 µm thick adhesive layer. In contrast, the earliest enamel fracture occurred in the model with 15 µm adhesive thickness.

Considering all restorative materials, dentin fracture occurred earliest in models with 15 µm adhesive thickness. For bulk-fill and conventional composite resins, the latest dentin fracture was observed in models with 10 µm adhesive thickness, while in the hybrid composite resin group, it was seen in models with 20 µm thick adhesive material.

Regarding the restorative materials themselves, the earliest fracture was found in models with 10 µm adhesive thickness for both bulk-fill and conventional composite resins. For hybrid composite resins, this occurred in 15 µm adhesive thickness. The latest fracture of the restorative material was noted in models with 20 µm thick adhesive material for both bulk-fill and hybrid composite resins. When hybrid composite resin was used, models with 15 µm thick adhesive layer demonstrated delayed fracture (Table 7).

When all adhesive thicknesses were compared, enamel fractures occurred earliest in restorations with bulk-fill composite resins. Hybrid composite resins showed the earliest onset of fracture within the restorative material, whereas bulk-fill composite resin demonstrated the most delayed restorative material fracture.

#### 3.2.2. Results of the Model with Class II Mesio-Occlusal Cavity

When Class II mesio-occlusal cavities were restored using bulk-fill, conventional, and hybrid composite resins and each material was evaluated individually, the shortest time to enamel fracture was observed in the model with a 10 µm thick adhesive layer. In contrast, the longest enamel lifespan was found in the model with 15 µm thick adhesive material. For dentin tissue, the earliest fracture occurred in the model with a 20 µm thick adhesive layer, while the latest result was seen in the model with 15 µm adhesive thickness. Regarding the restorative material, the fastest fracture was recorded in the model with 20 µm thick adhesive layer, whereas the slowest fracture occurred in the model with a 10 µm adhesive thickness (Table 8).

When all adhesive layer thicknesses were compared across all groups, the models restored with hybrid composite resin exhibited the longest lifespan for both enamel and dentin. In contrast, the earliest onset of fracture in these tissues occurred in models restored with bulk-fill composite resin. Additionally, the restorative material fractured earliest in the hybrid composite resin group and latest in the bulk-fill composite resin group.

#### 3.2.3. Results of the Model with Class II MOD Cavity

When Class II mesio-occluso-distal cavities were restored using bulk-fill, conventional, and hybrid composite resins and each material group was analyzed individually, the fastest enamel fracture occurred in the model with 10 µm thick adhesive layer. The slowest enamel fracture was observed in the model with 20 µm thick adhesive layer. For dentin, the earliest fracture was noted in the model with 20 µm adhesive thickness, whereas the longest survival was seen in the model with 15 µm adhesive thickness. The restorative material fractured fastest in the model with 15 µm thick adhesive material, while the most delayed fracture occurred in the model with 10 µm adhesive thickness (Table 9).

When all adhesive thicknesses were evaluated, hybrid composite resin restorations demonstrated the longest fracture resistance in both enamel and dentin. In contrast, bulk-fill composite resin restorations showed the earliest onset of failure in these tissues. Similarly, restorative materials fractured earliest in the hybrid composite resin group and latest in the bulk-fill composite resin group.

#### 3.2.4. Results of the Model with Class I Occlusal Cavity

When Class I occlusal cavities were restored with bulk-fill, conventional, and hybrid composite resins and each material was analyzed individually, the fastest enamel fracture was observed in the model with a 15 µm thick adhesive layer. The slowest enamel failure, indicating the longest lifespan, was recorded in the model with a 20 µm thick adhesive layer. For dentin, the earliest fracture occurred in the model with 20 µm thick adhesive material, while the longest-lasting performance was seen in the model with 15 µm adhesive thickness.

In restorations using conventional and hybrid composite resins, the latest restorative material fracture was observed in the model with 15 µm adhesive thickness, whereas the earliest occurred in the model with 20 µm thick adhesive material. For bulk-fill composite resin, the restorative material fractured earliest in the model with 10 µm thick and latest in the model with 20 µm thick adhesive material (Table 10).

When all adhesive thicknesses were assessed across all materials, hybrid composite resin restorations showed the longest enamel and dentin lifespan, while bulk-fill composite resin demonstrated the earliest tissue failure. For the restorative materials, fractures occurred sooner in hybrid composite resin and later in those restored with bulk-fill composite resin.

## 4. Discussion

The results of this study support the hypothesis that variations in the mechanical properties of restorative materials lead to differences in stress distribution within dental tissues and restorations, which in turn affect their fracture resistance.

The biomechanical response of teeth restored with Class I and Class II (MO, DO, MOD) cavity designs under vertical occlusal and fatigue loading has been widely explored through finite element analysis (FEA). While factors such as age and gender are known to influence the tooth’s resistance to masticatory forces, the specific impact of food texture and hardness, particularly in the mandibular first molar region, remains incompletely understood. During mastication, mandibular molars move across the opposing occlusal surfaces, crushing food and adapting its shape to the contours of the occlusal anatomy. The direction, magnitude, and application point of occlusal forces vary dynamically depending on the interaction between the food bolus and the tooth surface, as well as the physical properties of the food [31]. To replicate this complex loading behavior in the present study, compression-type contact elements were employed in a 3D finite element model. A consistent occlusal load of 600 N was applied in all simulations to ensure standardized comparison across conditions [32].

In the literature, different adhesive layer thicknesses are recommended: 2, 5, 10, and 30 μm. In a study by Ausiello et al. [14], a thin adhesive layer of 10 μm was used. Eliguzeloglu et al. [33] suggested that flexible materials such as glass ionomer cements, flowable composites, and nanofilled adhesives could help reduce stress under resin-based composite fillings. In the present study, adhesive layers of 10, 15, and 20 μm were chosen to examine the effect of adhesive material thickness on stress distribution, aiming to provide better flexibility. Numerous studies have been conducted on the long-term performance of these materials, and these studies ensure that survival rates are determined through long-term evaluations. Opdam et al. [34] emphasized that the patient’s caries risk plays a significant role in the longevity of restorations. Although amalgam has been the primary material for posterior restorations for many years, its use has decreased due to its inability to reinforce tooth structure and its potential to contribute to tooth fractures. With increasing aesthetic expectations, both dentists and patients are increasingly opting for tooth-colored materials as alternatives to amalgam in posterior restorations [19].

In restorative dentistry, a wide range of materials has been developed and continuously improved to enhance the mechanical strength and force resistance of dental tissues. A comprehensive understanding of oral biomechanics, combined with the application of this knowledge in the design and fabrication of restorations, plays a critical role in achieving long-term clinical success [35].

In a study conducted by Pietro Ausiello and colleagues, two types of bulk-fill resin composites and two different adhesive layer thicknesses were evaluated using finite element analysis on Class II MOD restorations. According to the findings of this study, models incorporating bulk-fill resin composites with a lower elastic modulus exhibited lower stress gradients compared to other models. This suggests that more flexible materials may have a superior capacity to absorb occlusal stresses more effectively. Furthermore, the study investigated the mechanical impact of varying adhesive bonding layer thicknesses. Across all loading scenarios, the thickness of the adhesive layer was found to have only a limited influence on the overall stress levels in restored teeth. These findings indicate that increasing the thickness of the adhesive layer did not significantly enhance its shock-absorbing properties. The results of our study are largely consistent with those reported by Ausiello et al. [36] In particular, our findings support the conclusion that materials with lower elastic moduli contribute to more favorable stress distribution patterns, and that adhesive layer thickness plays a minimal role in modifying stress concentrations. These insights emphasize the importance of selecting restorative materials not only based on aesthetic and strength considerations but also for their ability to modulate biomechanical behavior at the tooth–restoration interface. Such studies offer valuable scientific contributions toward the optimization of material selection and design strategies in restorative dentistry.

In 2003, Yaman et al. [37] performed a finite element analysis on a maxillary central incisor with a Class V cavity, using various brands of composite and compomer as restorative materials. Their findings demonstrated that the stress within the restored tooth structures was inversely related to the elastic modulus of the restorative materials. Consistent with their results, the present study showed that when bulk-fill composite resin—characterized by a low elastic modulus—was used, stress concentrations in the enamel and dentin were notably high. Similarly, the highest stress within the restorative material was observed when the material with the highest Young’s modulus was applied.

Yamanel et al. [4] conducted a finite element analysis to evaluate how different restorative materials and cavity designs influence stress distribution within both the tooth structure and the restoration itself. In their study, a nanofilled composite and two distinct full-ceramic materials were utilized. Their findings indicated that materials with lower elastic modulus values tended to transfer greater stress to the surrounding tooth tissues. These results align with those of the present study, in which full-ceramic inlay and onlay materials transmitted less stress to dental tissues compared to nanofilled composite materials.

In 2022, Gönder et al. [38] investigated stress distribution in teeth restored with varying adhesive layer thicknesses. Their findings demonstrated that increasing the adhesive thickness led to a reduction in stress accumulation within the adhesive layer—an outcome consistent with the present study. In a subsequent study published in 2023, the same group restored a molar tooth with different restorative materials following a Class II cavity preparation. Their results similarly showed that when materials with a lower Young’s modulus were used, higher stress levels were observed in the enamel and dentin, and fractures occurred earlier, supporting the trends identified in our findings [39].

In a study conducted by Ausiello et al. in 2001 [40], Class II MOD cavities were digitally prepared and restored in maxillary premolar teeth. Two composite resins were used as restorative materials: one with a high elastic modulus and the other with a low elastic modulus. The composite resin with a high elastic modulus caused greater stress accumulation at the enamel–dentin junction and the composite–tooth interface. Similarly, in our study, materials with a higher elastic modulus led to increased stress concentration within the restorative material itself, while lower-modulus materials transferred more stress to the surrounding dental tissues. These findings support the concept that the mechanical behavior of the restorative material plays a key role in the distribution of functional stresses, affecting both the restoration and the tooth structure.

The findings of the present study are supported by previous in vitro investigations that have demonstrated the significant impact of restorative material properties on the mechanical behavior of restored teeth. Belli et al., in 2005 [41], reported that different composite resins exhibit variable fracture resistance and failure modes under load, with conventional composite restorations showing lower resistance and more unfavorable fracture patterns. These results highlight the importance of mechanical characteristics such as elastic modulus and the material’s ability to absorb and distribute stress efficiently.

Similarly, Fonseca et al. [42] evaluated the performance of laboratory-processed composite resin restorations and found that, regardless of specific preparation design, all restored specimens showed significantly lower fracture resistance compared to sound teeth. Most failures involved both the restoration and the surrounding tooth structure, emphasizing the intrinsic mechanical limitations of composite resins even when processed under optimized laboratory conditions. These in vitro observations underline the critical role of material selection in achieving durable restorations and align with the mechanical trends observed in computational analyses.

There is evidence in the literature demonstrating that fatigue assessments based on finite element analysis (FEA) can yield results consistent with experimental data. Lin et al. [43] conducted both cyclic loading tests and finite element simulations on premolar teeth with longitudinal cracks, showing that the locations of crack initiation closely matched the high-stress areas predicted by FEA. Additionally, the fatigue life predictions derived from FEA-based S-N curves were in agreement with the actual fatigue lifespans observed in laboratory experiments. Similarly, Lohbauer et al. [44] reported that the mechanical fatigue degradation observed in resin-based composites follows comparable patterns in both laboratory settings and computational simulations. These findings support the notion that fatigue evaluations based solely on simulation, when properly constructed, can provide clinically relevant and valid results.

The finite element analysis (FEA) used in this study may not fully replicate real clinical conditions, and the results are independent of the biological variations present in vivo. The material properties applied in the analysis were modeled under ideal conditions, without accounting for intraoral factors such as temperature fluctuations, moisture, and long-term chemical interactions. Additionally, the study does not consider individual patient differences, including variations in chewing forces and biological adaptation, so caution is advised when generalizing the results. The analysis focused solely on stress generated by vertical occlusal loading, without evaluating the effects of lateral forces. Furthermore, changes in adhesive bonding performance over time and the long-term clinical behavior of restorations could not be directly assessed within this simulation.

Although finite element analysis reveals meaningful stress differences among materials and adhesive thicknesses, direct clinical thresholds for stress magnitudes are not yet well established. Nevertheless, higher stress concentrations—particularly at the adhesive interface—have been linked to increased risks of fatigue failure and marginal breakdown in previous studies [13,40]. Therefore, configurations showing lower stress values may be considered biomechanically more favorable, although further clinical correlation is needed.

## 5. Conclusions

The key findings of this study can be summarized as follows:

- The use of materials with a low Young’s modulus—indicating greater flexibility—resulted in significantly higher stress accumulation in the enamel and dentin, which, in turn, led to earlier fracture of these tissues. Conversely, stiffer materials with a higher Young’s modulus generated lower stress values in these regions. Thus, the stress in enamel and dentin was inversely related to the elastic modulus of the restorative material.
- Restorative materials with a higher Young’s modulus exhibited earlier structural failure compared to those with lower stiffness, indicating a trade-off between rigidity and internal stress tolerance.
- Increasing the thickness of the adhesive layer led to a reduction in stress accumulation within the adhesive itself, suggesting that a thicker adhesive layer may provide a protective buffering effect against stress concentration.

### Clinical Implications

In clinical practice, adhesive layer thickness cannot be precisely measured in microns. However, our results show that slightly increased adhesive thickness improves stress distribution under composite restorations. Therefore, although exact control is not possible, clinicians should avoid applying overly thin adhesive layers to minimize stress concentration and enhance restoration longevity.

## Figures and Tables

**Table 1:** Mechanical properties of dental tissues and restorative materials [19,20,21,22,23,24,25,26].

	Young’s Modulus (Mpa)	Poisson’s Ratio	Compressive Strength (MPa)	Flexural Strength (MPa)	Shear Strength (MPa)	Fracture Toughness (Mpa m^1/2^)	Microhardness (HV)
Enamel	84.1	0.33	384	11.5	60	0.8	3–6
Dentin	18.6	0.31	297	105.5	12–138	3.08	0.13–0.51
Adhesive Material	1	0.24	-	-	-	-	-
Pulp	0.002	0.45	-	-	-	-	-
PDL	0.0689	0.45	-	-	-	-	-
Cortical bone	13.7	0.3	-	-	-	-	-
Spongious bone	1.37	0.3	-	-	-	-	-
Bulk-fill Composite	12	0.25	169	42	-	-	-
Conventional Composite	16.6	0.24	294	77	-	-	-
Hybrid Composite	22	0.27	-	-	-	-	-

**Table 2:** Coefficient and exponent constants of fatigue curves of restorative materials and dental tissues [14,27,28,29,30].

Material	*A* (MPa)	*B*	*σ_f_* (MPa)	*b*
Enamel	-	-	310	−0.111
Dentin	-	-	247	−0.111
Bulk-Fill Composite	54	−0.020	-	-
Conventional Composite	84	−0.035	-	-
Hybrid Composite	182.8	−0.056	-	-

**Table 3:** Stress values (MPa) in dental tissues and restorative materials of the model with Class II disto-occlusal cavity.

	Model with 10 µm Thick Adhesive Material			Model with 15 µm Thick Adhesive Material			Model with 20 µm Thick Adhesive Material		
	Bulk-Fill Composite	Conventional Composite	Hybrid Composite	Bulk-Fill Composite	Conventional Composite	Hybrid Composite	Bulk-Fill Composite	Conventional Composite	Hybrid Composite
Enamel	44.53	43.47	44.33	61.14	59.67	58.83	44.98	44.86	44.88
Dentin	34.37	31.88	29.96	49.18	46.02	43.44	41.26	38.69	36.63
Restorative Material	19.24	20.99	22.27	17.93	18.51	19.60	16.96	18.52	19.61
Adhesive Material	17.29	14.83	13.08	10.24	10.04	10.06	9.981	9.938	9.854

**Table 4:** Stress values (MPa) in dental tissues and restorative materials of the model with Class II mesio-occlusal cavity.

	Model with 10 µm Thick Adhesive Material			Model with 15 µm Thick Adhesive Material			Model with 20 µm Thick Adhesive Material		
	Bulk-Fill Composite	Conventional Composite	Hybrid Composite	Bulk-Fill Composite	Conventional Composite	Hybrid Composite	Bulk-Fill Composite	Conventional Composite	Hybrid Composite
Enamel	69.06	67.20	65.86	61.26	59.50	58.24	63.21	61.56	60.39
Dentin	28.11	27.62	27.31	27.45	26.95	26.59	30.69	30.13	29.68
Restorative Material	14.70	15.90	16.84	14.80	16.21	17.18	15.55	16.66	17.47
Adhesive Material	18.35	16.08	14.20	9.377	7.946	6.766	8.388	7.096	6.282

**Table 5:** Stress values (MPa) in dental tissues and restorative materials of the model with Class II mesio-occluso-distal (MOD) cavity.

	Model with 10 µm Thick Adhesive Material			Model with 15 µm Thick Adhesive Material			Model with 20 µm Thick Adhesive Material		
	Bulk-Fill Composite	Conventional Composite	Hybrid Composite	Bulk-Fill Composite	Conventional Composite	Hybrid Composite	Bulk-Fill Composite	Conventional Composite	Hybrid Composite
Enamel	71.79	69.04	67.14	71.08	68.42	66.58	66.13	63.80	62.19
Dentin	22.73	22.71	22.70	22.50	22.48	22.47	22.86	22.84	22.82
Restorative Material	15.04	15.95	16.80	16.30	17.07	17.64	15.67	16.48	17.31
Adhesive Material	33.06	30.42	27.16	9.715	8.067	6.765	8.920	7.680	6.761

**Table 6:** Stress values (MPa) in dental tissues and restorative materials of the model with Class I occlusal cavity.

	Model with 10 µm Thick Adhesive Material			Model with 15 µm Thick Adhesive Material			Model with 20 µm Thick Adhesive Material		
	Bulk-Fill Composite	Conventional Composite	Hybrid Composite	Bulk-Fill Composite	Conventional Composite	Hybrid Composite	Bulk-Fill Composite	Conventional Composite	Hybrid Composite
Enamel	49.76	49.59	49.49	56.55	55.89	55.44	47.68	47.48	47.37
Dentin	31.07	30.56	30.13	30.80	30.35	29.98	35.17	34.54	33.99
Restorative Material	19.24	16.32	17.33	17.39	16.23	17.25	14.90	16.45	17.52
Adhesive Material	12.30	10.20	8.593	9.713	8.181	7.053	7.996	6.882	6.202

**Table 7:** The number of cycles required for fracture of dental tissues and restorative materials for the model with Class II disto-occlusal cavity.

	Model with 10 µm Thick Adhesive Material			Model with 15 µm Thick Adhesive Material			Model with 20 µm Thick Adhesive Material		
	Bulk-Fill Composite	Conventional Composite	Hybrid Composite	Bulk-Fill Composite	Conventional Composite	Hybrid Composite	Bulk-Fill Composite	Conventional Composite	Hybrid Composite
Enamel	5.382 × 10^9^	5.486 × 10^9^	5.528 × 10^9^	2.3768 × 10^8^	1.211 × 10^9^	3.474 × 10^8^	4.987 × 10^9^	5.095 × 10^9^	5.067 × 10^9^
Dentin	7.441 × 10^9^	1.5507 × 10^10^	2.830 × 10^9^	2.106 × 10^8^	4.088 × 10^8^	7.249 × 10^8^	1.210 × 10^9^	2.277 × 10^9^	3.889 × 10^9^
Restorative Material	5.921 × 10^37^	9.621 × 10^25^	6.378 × 10^21^	5.142 × 10^39^	6.529 × 10^27^	3.999 × 10^17^	7.867 × 10^40^	5.922 × 10^27^	9.016 × 10^22^

**Table 8:** Number of cycles required for fracture of dental tissues and restorative materials for the model with Class II mesio-occlusal cavity.

	Model with 10 µm Thick Adhesive Material			Model with 15 µm Thick Adhesive Material			Model with 20 µm Thick Adhesive Material		
	Bulk-Fill Composite	Conventional Composite	Hybrid Composite	Bulk-Fill Composite	Conventional Composite	Hybrid Composite	Bulk-Fill Composite	Conventional Composite	Hybrid Composite
Enamel	6.802 × 10^7^	8.976 × 10^7^	1.102 × 10^8^	2.280 × 10^8^	3.056 × 10^8^	3.776 × 10^8^	1.667 × 10^8^	2.167 × 10^8^	2.628 × 10^8^
Dentin	5.037 × 10^10^	5.959 × 10^10^	6.639 × 10^10^	6.325 × 10^10^	7.541 × 10^10^	8.575 × 10^10^	2.177 × 10^10^	2.599 × 10^10^	3.003 × 10^10^
Restorative Material	6.242 × 10^43^	3.132 × 10^29^	9.761 × 10^23^	5.345 × 10^44^	1.286 × 10^29^	5.976 × 10^23^	2.482 × 10^−42^	6.565 × 10^28^	4.629 × 10^23^

**Table 9:** The number of cycles required for fracture of dental tissues and restorative materials in the model with a Class II mesio-occluso-distal (MOD) cavity.

	Model with 10 µm Thick Adhesive Material			Model with 15 µm Thick Adhesive Material			Model with 20 µm Thick Adhesive Material		
	Bulk-Fill Composite	Conventional Composite	Hybrid Composite	Bulk-Fill Composite	Conventional Composite	Hybrid Composite	Bulk-Fill Composite	Conventional Composite	Hybrid Composite
Enamel	4.657 × 10^7^	6.962 × 10^7^	9.291 × 10^7^	5.132 × 10^7^	7.553 × 10^7^	9.955 × 10^7^	1.075 × 10^8^	1.546 × 10^8^	2.003 × 10^8^
Dentin	3.853 × 10^11^	3.882 × 10^11^	3.897 × 10^11^	4.218 × 10^11^	4.250 × 10^11^	4.266 × 10^11^	3.632 × 10^11^	3.659 × 10^11^	3.688 × 10^11^
Restorative Material	1.502 × 10^43^	2.254 × 10^29^	9.235 × 10^23^	2.089 × 10^41^	3.360 × 10^28^	4.022 × 10^23^	1.404 × 10^42^	8.768 × 10^28^	5.504 × 10^23^

**Table 10:** Number of cycles required for fracture of dental tissues and restorative materials of the model with Class I occlusal cavity.

	Model with 10 µm Thick Adhesive Material			Model with 15 µm Thick Adhesive Material			Model with 20 µm Thick Adhesive Material		
	Bulk-Fill Composite	Conventional Composite	Hybrid Composite	Bulk-Fill Composite	Conventional Composite	Hybrid Composite	Bulk-Fill Composite	Conventional Composite	Hybrid Composite
Enamel	1.817 × 10^9^	1.878 × 10^9^	1.916 × 10^9^	5.052 × 10^8^	5.686 × 10^8^	6.189 × 10^8^	2.742 × 10^9^	2.858 × 10^9^	2.924 × 10^9^
Dentin	1.918 × 10^10^	2.250 × 10^10^	2.578 × 10^10^	2.096 × 10^10^	2.415 × 10^10^	2.718 × 10^10^	5.759 × 10^9^	6.864 × 10^9^	8.022 × 10^9^
Restorative Material	4.278 × 10^37^	1.130 × 10^29^	5.078 × 10^23^	6.939 × 10^39^	1.349 × 10^29^	5.588 × 10^23^	1.744 × 10^43^	9.177 × 10^28^	4.195 × 10^23^

## Data Availability

The original contributions presented in this study are included in the article. Further inquiries can be directed to the corresponding author.

## Abbreviations

DICOM	Digital Imaging and Communications in Medicine
STL	Standard Tessellation Language
FEA	Finite Element Analysis
CBCT	Cone-Beam Computed Tomography
Gpa	Gigapascal
Mpa	Megapascal
